# Rowell's American Newspaper Directory

**Published:** 1880-10

**Authors:** 


*American Newspaper Directory.* 1880. Publishers: Geo. P. Rowell & Co., New York.

This work which reflects credit upon the enterprising publishers, contains accurate lists of all the newspapers and periodicals published in the United States, Territories, and Dominion of Canada, together with a description of the towns and cities where published.

